# Nuclear protein IK undergoes dynamic subcellular translocation and forms unique nuclear bodies during the cell cycle

**DOI:** 10.1186/2050-7771-1-11

**Published:** 2013-02-18

**Authors:** Liyan Hu, Feikun Yang, Xianan Liu, Dazhong Xu, Wei Dai

**Affiliations:** 1 Department of Environmental Medicine, New York University Langone Medical Center, Tuxedo, NY, 10987, USA; 2 Department of Biochemistry & Molecular Pharmacology, New York University Langone Medical Center, Tuxedo, NY, 10987, USA

## Abstract

IK is a nuclear protein containing a unique domain named RED due to the presence of a repetitive arginine (R), aspartic (E), and glutamic acid (D) sequence. To date, the function of this protein remains largely unknown despite of a couple of previous studies in the literature. Here we report that depletion of IK via RNA interference results in mitotic arrest. We also demonstrate that IK undergoes dynamic translocation during interphase and mitosis. In particular, IK is primarily present in some interphase cells as nuclear foci/bodies which do not co-localize with nucleoli, PMA bodies and Cajal bodies. Pull-down analysis coupled with mass spectrometry reveals that IK is associated with DHX15, a putative ATP-dependent RNA helicase. Our results strongly suggest that IK may participate in pre-mRNA splicing and that it may be a useful biomarker for a new nuclear structure in the cell.

## Introduction

IK was originally identified as a cytokine that inhibits interferon gamma (IFN-γ)-induced expression of HLA class II antigen during immune responses in K562 erythroleukemic cell line [1]. The protein got its name IK based on these findings. Human IK contains 557 amino acids and migrates at about 80 kDa on SDS-PAGE [2]. It is also named RED owing to the presence of a repetitive arginine (R), aspartic (E), and glutamic acid (D) sequence [2]. It has also been reported that IK is one of the spliceosome factors [3,4]. Screening of an siRNA library containing 23,835 human genes reveals that depletion of IK induces mitotic arrest, primarily characterized by having a high mitotic index [5]. A recent study shows that IK is required for the localization of MAD1, a spindle checkpoint protein, to the kinetochores and involved in the regulation of the spindle assembly checkpoint [6].

In the present study, we have confirmed that depletion of IK causes mitotic arrest. Our further investigation reveals that the subcellular localization of IK is dynamic during the cell cycle. We also show that the expression of IK is cell cycle-regulated. Affinity pull-down and mass spectrometry analyses reveal that IK interacts with DHX15, a putative ATP-dependent RNA helicase which is implicated in pre-mRNA splicing. Our current study suggests that IK can be explored as a new biomarker for cell proliferation and checkpoint control.

### Materials and methods

#### Cell culture

HeLa cell line was originally obtained from the American Type Culture Collection (Manassas, VA). Cells were cultured in DMEM supplemented with 10% fetal bovine serum (FBS, Invitrogen, Carlsbad, CA) and antibiotics (100 μg/ml of penicillin and 50 μg/ml of streptomycin sulfate, Invitrogen) at 37°C under 5% CO_2_.

#### Antibodies and plasmids

Antibodies for IK was purchased from Bethyl Laboratory Inc (Montgomery, TX). GFP antibody was purchased from Santa Cruz Biotechnology (Santa Cruz, CA). Coilin antibody was purchased from Abcam (Cambridge, MA). GFP-IK and His_6_-IK were subcloned as described in a previous study [7].

#### RNA interference

IK small interfering RNAs (IK siRNA) was purchased from Dharmacon, which corresponds to following sequences: NNCAUAUGAGCGGAAUGAGUU. HeLa cells were seeded at 50% confluence in an antibiotic-free culture medium and transfected with siRNAs at a final concentration of 10 nM for 24, 48, or 72 h using the LipoJet™ In Vitro Transfection Kit (Ver. II, Signagen Laboratories, Rockville, MD). Negative controls were cells transfected with 10 nM siRNAs targeting firely (*Photinus pyralis*) luciferase. The sequence of the control siRNA is 5’UUCCTACGCTGAGTACTTCGA3’ (GL-2, Dharmacon, Waltham, MA).

#### Western blot

SDS-PAGE was carried out using the mini gel system from Bio-Rad (Hercules, CA). Proteins were transferred to PVDF membranes. After blocking with TBST containing 5% nonfat dry milk for 1 h, the membrane was incubated overnight with a primary antibody, followed by incubation with a horse reddish peroxidase-conjugated secondary antibody for 1 h at room temperature. After thorough washing with TBST buffer, signals on the membranes were developed with an enhanced chemiluminescent system (Pierce, Rockford, IL).

#### Immunoprecipitation and pull-down assay

Immunoprecipitation and pull-down assay were performed as described previously [6,7]. For pull down assay, HEK293K cells transfected with His_6_-IK were lysed in a lysis buffer (50 mM sodium phosphate, 300 mM sodium chloride, 20 mM imidazole, 1% NP40, pH 7.4). Ni^2+^ -IDA-agarose resins (Clonetech, Mountain View, CA) were then added to the cell lysates and incubated with gentle agitation at 4°C overnight. The resin was then washed three times with the washing buffer [50 mM sodium phosphate, 300 mM sodium chloride, 40 mM imidazole (pH 7.4)]. After last wash, His_6_-tagged products were eluted in the elution buffer (50 mM sodium phosphate, 300 mM sodium chloride, 300 mM imidazole, pH 7.4). The elutes were diluted in a lyses buffer [20 mM Tris (pH 7.5), 150 mM NaCl, 1% Triton, 2 mM sodium pyrophosphate, 1 mM EDTA, 1 mM NaF, 1 mM sodium orthovanadate, 500 μM PMSF, 2 μM pepstatin A, 10 units/ml aprotinin, 20 mM NEM] and cleared by centrifugation. The anti-IK antibody or control IgG (1 μg) as well as protein G-agarose (40 μl) resins (50/50, Millipore, Billerica, MA) were then added to cell lysates and incubated at 4°C overnight. The resins were then extensively washed with the lysis buffer. Proteins bound to the resin were eluted with SDS sample buffer and then subjected to SDS-PAGE followed by Western blot with appropriate antibodies.

#### Fluorescence microscopy

Fluorescence microscopy was performed as described in our previous studies [8]. Briefly, HeLa cells seeded on chamber slides were transfected with various expression constructs for 48 h. At the end of transfection, cells were fixed with 4% paraformaldehyde in PBS for 20 min at room temperature. After permeabilization by incubation with 0.5% Triton X100 in PBS for 20 min, cells were incubated with 2% bovine serum albumin in PBS for 1 h followed by incubating overnight with the antibody to GFP. Cells were stained with Alexa Fluor 488. Cellular DNA was finally stained with 4’, 6-diamidino-2-phenylindole (DAPI, Molecular Probe, Eugene, OR). Fluorescence signals were detected on a Leica TCS SP5 confocal microscope or on a Leica AF6000 fluorescence microscope.

#### Flow cytometry

Flow cytometry was performed as described in our early studies [9]. Briefly, cells were initially fixed in 75% ethanol, then suspended in a solution of PBS containing 100 μg/ml of RNase A (Sigma, St Louis, MO) and 10 μg/ml of propidium iodide (Molecular probes, Eugene, OR) and kept at room temperature for 1 h. Cellular fluorescence was then measured using Beckman Coulter Epics XL-MCLTM Flow Cytometer (Fullerton, CA). DNA content was deconvoluted using Muticycle software (Phoenix Flow System, San Diego, CA) to estimate percent of cells in different phases of the cell cycle.

#### Statistical analysis

Student’s *t* test was used to evaluate the difference between two groups. The significant level was set at 0.05.

## Results

**Figure 1:**
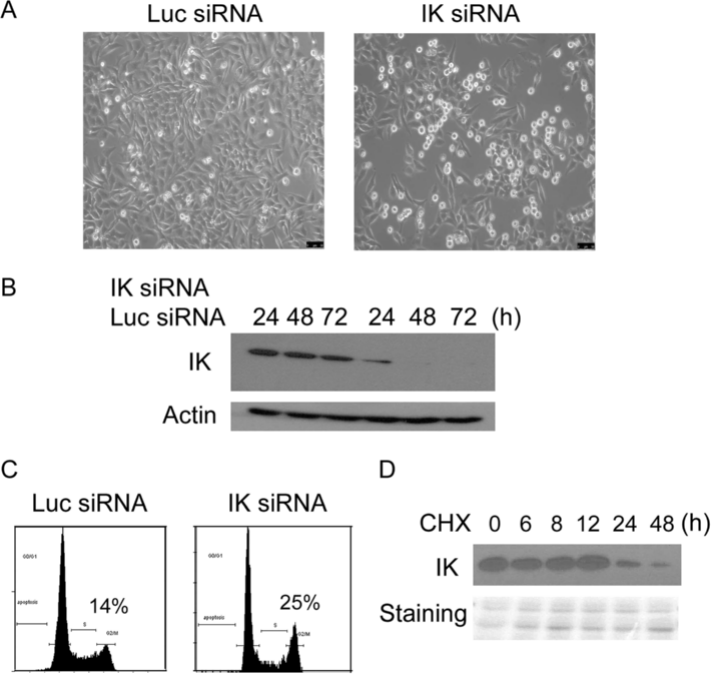
**IK is a stable protein required for cell cycle progression. A**. HeLa cells were transfected with either control siRNA (targeting luciferase) or IK siRNA. Forty eight hours after transfection, cells were photographed under a phase-contrast microscope. **B**. HeLa cells were transfected with IK siRNA. IK protein levels were followed at indicated time points by Western blot with an anti-IK antibody. **C**. HeLa cells were transfected as in A and DNA content of the cells were analyzed by flow cytometry 48 hours after transfection. **D**. Half-life analysis of IK protein was determined after treatment with cycloheximide (CHX) followed by Western blot with the anti-IK antibody.

A previous study showed that IK might be involved in the cell cycle regulation because its depletion resulted in apparent mitotic arrest [5]. To confirm its role during cell cycle regulation, we transfected HeLa cells with IK siRNA or luciferase siRNA as control. Transfection with IK siRNA for 48 h induced a significant increase in cells with a rounded-up phenotype (Figure 1A), suggesting mitotic arrest. Blotting with an IK specific antibody revealed that knocking down was efficient (Figure 1B). DNA content analysis by flow cytometry also showed an increase in G_2_/M population after transfection with IK siRNA (Figure 1C). Given that there was no significant increase in rounded-up cells 24 h after transfection with IK siRNA (data not shown), we suspected that IK protein had a relatively long half-life. Blocking new protein synthesis after treatment with cycloheximide (CHX) followed by immunoblotting revealed that significant decrease in IK protein levels occurred 24 h after the treatment (Figure 1D), indicating that IK does have a relatively long half-life.

**Figure 2:**
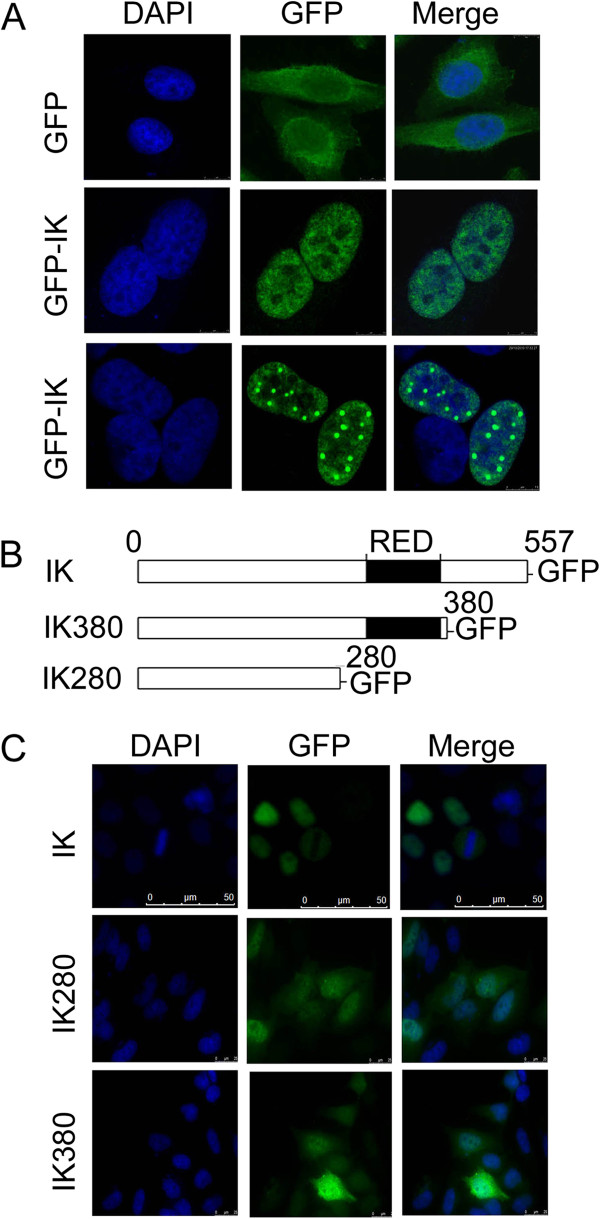
**Ectopically expressed IK proteins form nuclear foci during interphase, a process that requires its C-terminal region. A**. HeLa cells were transfected with a plasmid expressing GFP or GFP-IK. Expression of GFP or GFP-IK was examined under a fluorescence microscope as described in Materials and methods. **B**. Schematic representation of the IK deletion constructs used in this experiment. **C**. IK deletion mutants were unable to form nuclear foci and present in both the nucleus and the cytoplasm during interphase. HeLa cells were transfected with indicated IK constructs and expressed proteins were examined under a fluorescence microscope as described in Materials and methods.

To understand the role of IK during the cell cycle, we first studied its subcellular localization during the cell cycle. HeLa cells were transfected with a plasmid construct expressing GFP-tagged IK, or GFP as a control, for 48 hours and processed for fluorescence microscopy. Ectopically expressed GFP-IK signals were exclusively located in the nuclei (Figure 2A). Detailed analysis revealed that GFP-IK exhibited two distinct types of localization patterns. A majority of cells contained GFP-IK signals that were distributed evenly across the nucleus (Figure 2A). On the other hand, GFP-IK signals also formed round nuclear bodies of difference sizes and the number of nuclear bodies varied from 2–30 per cell (Figure 2A, lower panel). To quantify the percentage of cells showing IK nuclear bodies, HeLa cells were transfected with the plasmid expressing GFP-IK for 48 hours and then fixed for immunostaining with an antibody to GFP. Among the GFP-IK positive cells, about 8% showed nuclear speckles (data not shown)

Since IK contains a distinct RED domain [2], we speculated that this domain may have a significant role in regulating IK localization, as well as its function. We made two plasmid constructs containing GFP-tagged deletion mutants of IK (IK280 and IK380). IK280 contains the N-terminal region of IK without the RED domain and the C-terminal region next to the RED domain whereas IK380 has the RED domain but without the C-terminal region (Figure 2B). We transfected these and the wild type IK constructs into HeLa cells. We found that neither IK280 nor IK380 displayed nuclear foci/bodies when they were expressed in HeLa cells (Figure 2C). Moreover, a significant amount of IK280 and IK380 was found in the cytoplasm, which is in contrast to the unique nuclear localization of the full length GFP-IK. These observations suggest that the C-terminal region next to the RED domain (amino acids 380–557) is required for the nuclear localization and the formation of nuclear speckles. The RED domain may or may not be required for these processes.

**Figure 3:**
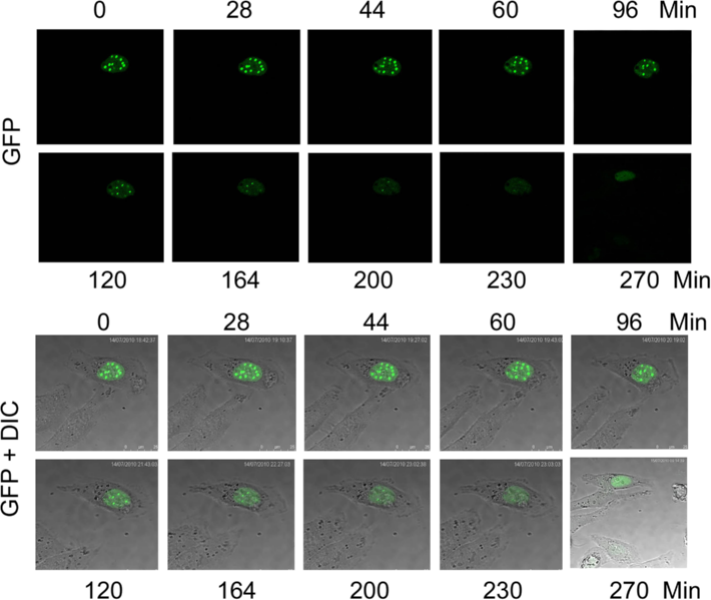
**Formation of IK nuclear foci is dynamic.** HeLa cells were transfected with the full-length IK-GFP construct. GFP-IK nuclear signals were followed by time-lapse microscopy under a confocal microscope.

Since IK was present as nuclear foci/bodies only in a small population of interphase cells, we speculated that the subcellular localization of IK might be regulated during the cell cycle. To test this possibility, we used time-lapse confocal microscopy to examine the formation of nuclear speckles in HeLa cells ectopically expressing GFP-tagged IK. We observed that IK nuclear body gradually appeared and became more condensed over time during cell cycle progression (Figure 3A, Arrow). Eventually, all nuclear foci/bodies disappeared and GFP-IK signals spread evenly in the nucleus. These observations suggest that the formation of IK foci/bodies is a dynamic process during the interphase.

**Figure 4:**
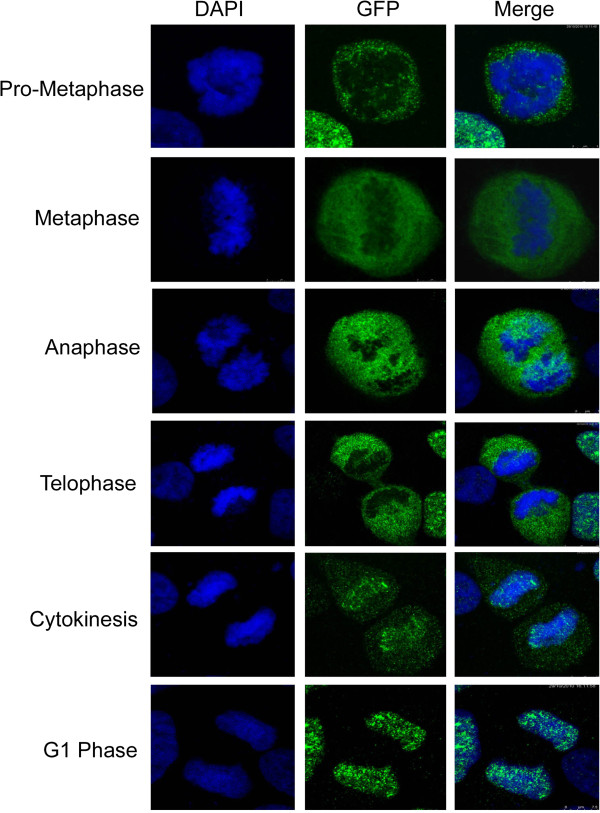
**Dynamic localization of IK protein during mitosis.** HeLa cells were transfected with a plasmid expressing GFP-tagged full-length IK. GFP-IK signals in cells of different mitotic stages were examined by fluorescence microscopy as described in Materials and methods.

Given that knocking down of IK induces mitotic arrest, we next examined its subcellular localization during mitosis. GFP-IK was detectable throughout mitosis (Figure 4). During prometaphase, metaphase, and anaphase, GFP-IK signals were mainly confined to in the nucleoplasm and chromosomes were largely free of IK signals. In telophase, GFP-IK signals started to accumulate on the chromosomes. In newly divided daughter cells (G_1_), almost all IK signals were present on chromosomes, overlapping with DAPI signals. Again, these results strongly suggest that the subcellular localization of IK is precisely regulated during the cell cycle.

**Figure 5:**
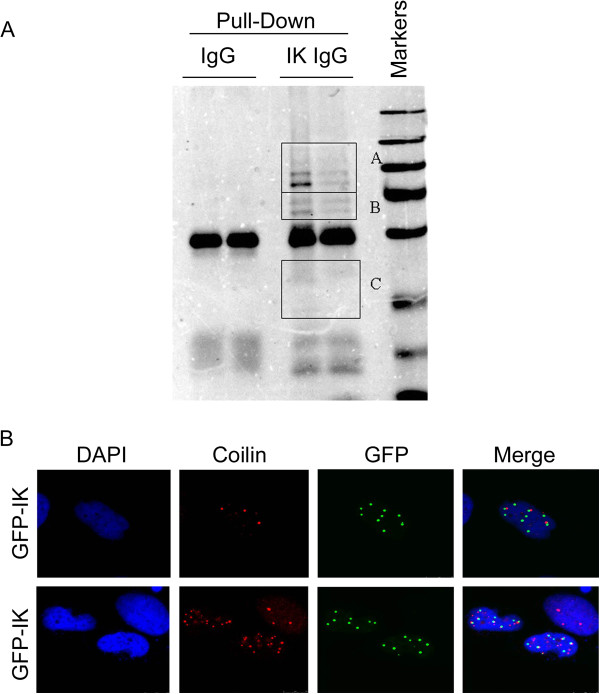
**Identification of IK binding proteins. A**. A two-step pull-down procedure followed by SDS-PAGE was carried out as described in Materials and methods. The 3 squares indicate proteins in the areas that were subjected to analysis with mass spectrometry. **B**. HeLa cells were transfected with the GFP-IK expressing construct for 2 days. Cells were then fixed and stained with antibodies to GFP and coilin, a Cajal body component, and specific signals were examined by fluorescence microscopy.

To understand the function of IK, we sought to identify proteins that interact with IK. We performed affinity enrichment experiments in which ectopically expressed His_6_-IK, along with its interacting proteins, was pulled down by Ni-IDA resins followed by immunoprecipitation with the anti-IK antibody. Immunoprecipitates were fractionated by SDS-PAGE followed by staining with Coomassie Blue. Compared to the control IgG, the IK IgG specifically precipitated several unique proteins (Figure 5A). Mass spectrometry analysis identified a series of new proteins that may potentially interact with IKs (Table 1). Among the major proteins that interacted with IK is DHX15, a putative ATP-dependent RNA helicase implicated in pre-mRNA splicing. Because Cajal bodies contain pre-mRNA splicing factors and several snRNPs [10-12], we reasoned that IK bodies may co-localize with Cajal bodies. To test this possibility, HeLa cells transfected with GFP-IK expression plasmid were fixed and stained with antibodies to GFP and coilin, the latter being a component of Cajal bodies. Fluorescent microscopy revealed that GFP-IK did not co-localize with coilin very well (Figure 5C), suggesting that IK bodies may be different from cajal bodies.

**Table 1:** IK-associated proteins identified by mass spectrometry

	
1	IK Protein RED
2	DHX15 Putative pre-mRNA-splicing factor ATP-dependent RNA helicase DHX15
3	HSPA1A;HSPA1B Heat shock 70 kDa protein 1A/1B
4	ZNF518B Zinc finger protein 518B
5	ZSCAN5B Zinc finger and SCAN domain-containing protein 5B
6	SYTL2 Isoform 2 of Synaptotagmin-like protein 2
7	WNT3 Proto-oncogene Wnt-3
8	IL25 Isoform 1 of Interleukin-25
9	ZNF711 Isoform 3 of Zinc Finger Protein 711
10	PRKRIR Isoform Long of 52KD repressor of the inhibitor of the protein kinase
11	LAMB1 Laminin subunit beta-1

## Discussion

In this study, we have shown that extended depletion of IK induces mitotic arrest, which is consistent with an early report [5]. IK is a relatively stable protein with a half life about 24 h, which may explain the delayed mitotic arrest after IK depletion. On the other hand, IK-induced mitotic arrest can be an indirect effect due to affecting the splicing of key cellular targets involved in cell division.

Affinity pull-down and mass spectrometry identify many new proteins interacting with IK. The association of IK with DHX15, a spliceosome factor with a RNA helicase activity, is consistent with the previous observation that IK is a member of the spliceosome [3,4]. Interestingly, we find that IK does not co-localize with coilin, a main component of the Cajal body (Figure 5B). As IK bodies do not co-localize with nucleoli and PML bodies, these signals may represent a novel nuclear structure. It has been reported that DHX15 directly interacts with Tuftelin-interacting protein (TFIP11), a component of the spliceosome, to promote the release of the lariat-intron during late-stage splicing [13,14]. TFIP11 has been shown to reside in a structure termed as TFIP11 bodies outside of nucleoli, which are distinct from cajal bodies, PML body, splicing speckles (SC35), paraspeckles, snRNP, hnRNP, or perinucleolar compartment [15,16]. Thus, it is worthwhile to determine whether IK may also localize to TFIP11 bodies.

The dynamic translocation of IK signals during interphase and mitosis is intriguing. It has been recently reported that IK is a spindle pole-associated protein that colocalizes with MAD1 at spindle poles during metaphase and anaphase [6]. Further studies will be focused on elucidating the relationship between subcellular translocation with its function. It is interesting that the RED domain along does not affect the formation of nuclear bodies and nuclear localization of IK. However, we can not rule out that both the RED domain and the C-terminal region of IK are required for these processes. Further experiments need to be done to address this question. Nonetheless, the unique RED domain is likely to have some functional role. Future study will explore this possibility.

In summary, the current study indicates that IK may play an important role during the cell cycle as depletion of IK induced mitotic arrest. Moreover, the subcellular localization of IK is dynamically regulated during the cell cycle. Furthermore, we identify that IK interact with pre-mRNA helicase DHX15, a spliceosome component. Although the association of IK with the spliceosome has been reported [3,4], this is the first report, to our knowledge, to show the physical association between IK and DHX15. It is likely that regulation of cell cycle progression by IK may also work through controlling mRNA splicing of genes important mitotic progression.

## Competing interests

All authors declare that they have no competing interests.

## Authors’ contributions

LYH designed various experiments, carried out the studies, and drafted the manuscript. FKY was involved in designing some of the experiments. XNL helped in making certain expressing plasmid constructs. DZX helped in drafted the manuscript and was involved in data interpretation and discussions. WD was involved in designing experiments, data interpretations, and writing the manuscript. All authors have read and approved the final manuscript.
